# Automated shape-transformable self-solar-tracking tessellated crystalline Si solar cells using in-situ shape-memory-alloy actuation

**DOI:** 10.1038/s41598-022-05466-7

**Published:** 2022-01-31

**Authors:** Min Ju Yun, Yeon Hyang Sim, Dong Yoon Lee, Seung I. Cha

**Affiliations:** 1 Energy Conversion Research Center, Electrical Materials Research Division, Korea Electrotechnology Research Institute, Changwon, Korea; 2 Department of Electro-Functionality Materials Engineering, University of Science and Technology, Changwon, Korea

**Keywords:** Energy harvesting, Photovoltaics

## Abstract

Photovoltaic energy systems in urban situations need to achieve both high electricity production and high capacity in restricted installation areas. To maximize power output, solar-tracking systems tilt solar arrays to track the sun’s position, and typically flat modules are used to maximize the cross-sectional area. Such tracking systems are complex and expensive, and flat modules cannot utilize omnidirectional incident light. For solar systems in urban environments, we have developed two-dimensional (2D) or three-dimensional (3D) tessellated solar-cell modules that use shape transformation, and combine solar tracking and an arch structure for use in restricted areas. The modules can use scattered and omnidirectional incident light. Simply by attaching shape-memory alloy strips to the surface of the solar panels, the shape of the array can be transformed in response to heat from sunlight. Compared to a perfect solar-tracking system, our simulation results indicate that the modules present a large cross-sectional area perpendicular to the direction of sunlight and provide superior tracking performance, resulting in a 60% increase in electricity production over the course of 1 day. In addition, by using different designs for the tessellation units, dome shaped or other 3D structures are possible, for specific applications and to meet aesthetic requirements.

## Introduction

The use of photovoltaic (PV) technology as a renewable energy source has expanded in recent years^1–4^. For PV generation to be applied in urban settings, high electricity production with sufficient capacity is necessary. Typically, the generation performance of fixed PV modules diminishes when the surface of the PV cell is not aligned perpendicular to the direction of sunlight. To prevent such losses and maximize power output, active or passive solar-tracking systems are conventionally used with fixed PV modules so that flat PV modules are tilted to track the position of the sun over the course of a day^5–13^. The key function of a solar-tracking system is to maximize the cross-sectional area incident to sunlight, maintaining the PV cell surface perpendicular to incident sunlight to maximize efficiency. However, such tracking systems are complex and introduce moving machinery that is expensive, occupies more space and requires more maintenance. These requirements are particularly challenging in urban areas, as the introduction of heavy additional machinery and a wider swing area is nearly impossible in building-integrated PV cells, or cells in space-restricted areas. For arrays of modules, the resulting shadow becomes so long that a wide space between modules is required for proper operation, which presents an additional barrier to urban application of multiple modules.

For urban applications of PV cells, it is important to utilize not only direct incident light but also omnidirectional light within a restricted installation area. Typically, solar cells or PV panels have flat or two-dimensional (2D) geometry to provide a wider cross-sectional area and receive uniform incident light to avoid mismatch loss. However, several recent studies have shown that 2D or 3-dimensional (3D) solar-cell arrays with more complex cylindrical, fractal structures or prism concept can receive omnidirectional light and produce more electricity within a smaller installation area^14–20^.

In urban environments, to maximize electricity production, a combination of solar tracking and 2D or 3D structured solar cells or modules should be applied. In this study, we introduce a shape-transformable self-solar-tracking tessellated solar cell array that uses shape-memory-alloy components as actuators to automatically change the shape of the array in response to the sun’s position, based on changes in the surface temperature of the cells. The cross-sectional area perpendicular to the incident light increases as the actuator flattens the panels, facilitating an automated solar-tracking effect. In addition, this solar-tracking concept can be applied to tessellated modules, which have the advantage that they use widely available commercial crystalline silicon (Si) solar cells.

Shape-transformable 3D modules could be applied in both urban settings and rural environments. This approach can produce the same or slightly higher amounts of electricity through all angles of incidence (AOIs) by active shape deformation, and provides a 60% increase in electricity production over a day compared to a fixed flat panel. In addition, electricity generation from arrays is further enhanced by the shorter shadow length. A bifacial effect is obtained during shape transformation, in which direct light is gathered effectively on some surfaces and scattered and reflected light is gathered on other surfaces, an effect that cannot be obtained in solar modules that use conventional tracking systems.

The performance of the new solar panel concept exceeds that of existing tracking solar panels. A very simple arch geometry is demonstrated here, but other geometries will be investigated and optimized in future work. This concept provides a revolutionary approach for future PV applications, particularly in urban environments with restricted installation areas.

## Results

**Figure 1:**
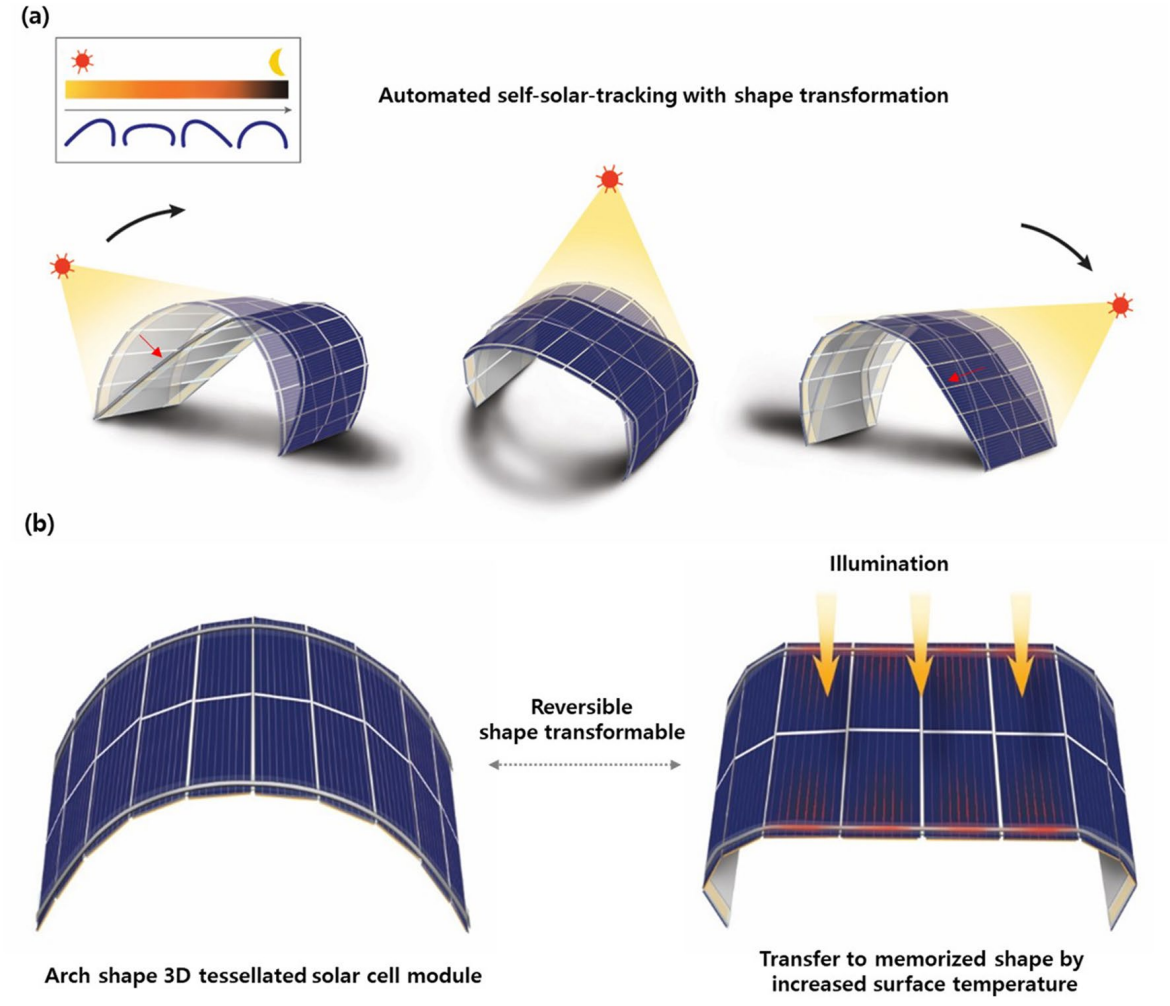
(**a**) Schematic illustration of the concept of automated self-solar-tracking using a shape-transformation 2D tessellated solar cell array during 1 day. (**b**) Schematic illustration of the mechanism of reversible shape transformation via transfer of the heat of the solar-cell surface induced by sunlight.

In conventional solar tracking, a flat PV panel is moved according to the movement of the sun so that the panel remains perpendicular to incident sunlight. Theoretically, electricity generation from a flat rigid panel will be optimized if the sun is tracked perfectly. However, conventional solar tracking has significant drawbacks, such as the need for machinery, additional energy consumption, increased shadow, and cost; it is therefore difficult to utilize these systems in urban environments with a restricted installation area, or in building-integrated applications. To obtain similar or superior results to those of conventional solar-tracking systems while addressing these limitations, the concept of a shape-transformable 3D solar cell array is proposed here, as shown in Fig. 1a. Shape-transformable 3D solar cell arrays change shape according to the sun’s position, inducing a maximized incident light cross-sectional area by maintaining the solar-cell surface perpendicular to the incident sunlight. Shape transformation is achieved without additional mechanical parts simply by attaching shape-memory-alloy components to the system, which are reversibly actuated by transfer of the heat induced by sunlight incident to the surface of the solar cells. Even a simple 2D arch design can demonstrate solar-tracking effects, and we expect to be able to extend the concept to more complex structures while maintaining the underlying principles, thereby creating more efficient PV systems.

The shape-transformable 2D solar cell arrays demonstrated here were based on tessellated wafer-based mono-crystalline Si solar cells. The tessellated structures can be constructed from small solar-cell units that come in a range of shapes such as rectangles, equilateral triangles and right-angled triangles. An elastic material such as silicone rubber or a metal mesh should be used as the backbone to maintain a 2D arch shape and to allow recovery of the original shape from the transformed shape. The individual small solar-cell units are arranged on the backbone strip at regular intervals and can be electrically connected using metal wire or textile electrodes and soldering. Shape-memory-alloy strips are attached to the surface of each solar cell panel and then the tessellated solar cells are encapsulated in a silicone material using a casing method. The overall shape and possible transformable shapes are determined by the morphology of the tessellated units and the geometry of the linkages. Shape-transformation can be obtained by using shape-memory-alloy components that have memorized a flat structure, as shown in Fig. 1b. When the surface temperature of the solar cell is increased by sunlight, the shape-memory alloy recovers to a programmed memorized flat shape, hence actuating the system. The flat memorized shape means that the surface of the tessellated solar cell will maximize the cross-sectional area incident to sunlight.

**Figure 2:**
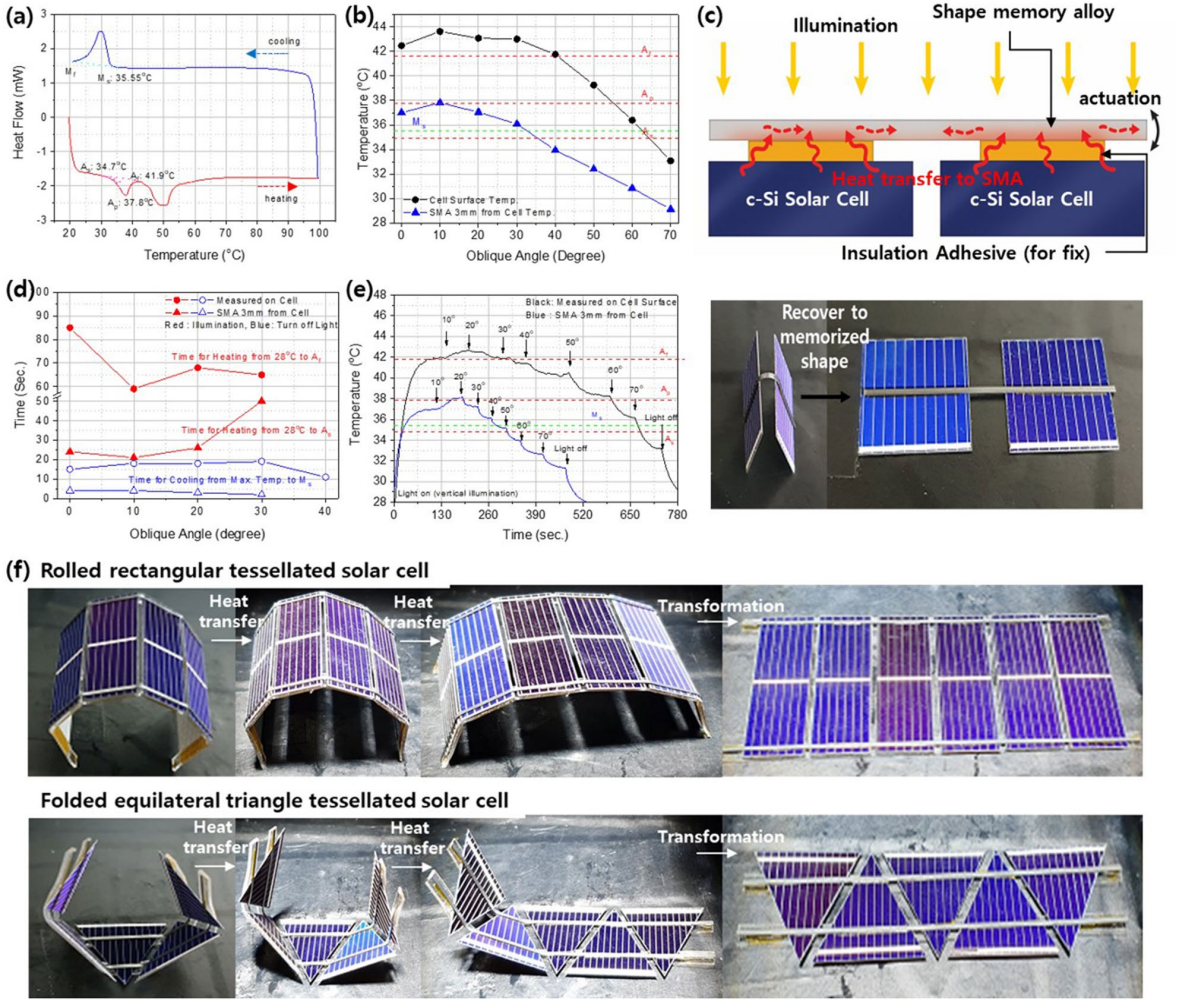
(**a**) Differential scanning calorimetry (DSC) analysis of shape memory and (**b**) the temperatures of the surface of the solar cell and the shape-memory alloy attached to the solar cell. (**c**) Schematic illustration of the heat-transfer process and shape-memory actuation. (**d**,**e**) Heating and cooling times of the surface of the solar cell and of the shape-memory alloy depending on the angle of incidence (AOI) under illuminated and non-illuminated conditions. (**f**) Photographs of the transformation process of rectangular and equilateral-triangle tessellated solar cells from folded to flattened shapes.

The shape-memory-alloy components are transformed by an increase in temperature on the surface of the solar cell in response to illumination with sunlight. Therefore, the temperature changes that result from changes in the AOI, and the shape changes that arise in response to changes in the temperature of the solar-cell surface should be analyzed. In this study, a nickel titanium shape-memory alloy was used (NiTi; the most widely used shape-memory alloy), with an austenite transition starting at 34.7 °C (As) and finishing at 41.9 °C (Af) in DSC analysis, as shown in Fig. 2a. The martensite transition starts at 35.5 °C (Ms) during cooling. Therefore, at temperatures of less than 34.7 °C, the shape-memory alloy is in the relatively flexible martensite phase, and above that temperature the alloy stiffens and returns to the memorized shape, with this phase transition ending at 41.9 °C on heating. On cooling to below the Ms temperature, the alloy returns to its original free-to-deform state. The phase-transition temperature can be controlled by the composition stoichiometry to within a suitable temperature range, which differs depending on the application. The temperature of a passivated emitter rear cell (PERC)-type crystalline Si solar cell differs with the AOI under illumination conditions of 1 sun, 1.5 A.M., as shown in Fig. 2b. Considering the austenite transition temperature, an AOI of less than 60° provides sufficient temperature for the phase transition or shape-memory-alloy shape changes. However, in the tessellated modules, transformation of the solar-cell array is induced by actuation of the shape-memory-alloy components between the tessellation units and in the linkage spaces, as shown in Fig. 2c. Therefore, the temperature of the shape-memory-alloy components between the solar-cell surfaces is more important than the temperature of the surfaces themselves. The temperatures of the shape-memory-alloy components located between the solar-cell units at a distance of 3 mm from the cell surface and that of the linkage backbone follow a similar trend to the temperature of the solar-cell surface, but with values that are 2–6 °C lower. Therefore, an AOI of less than 30° can provide sufficient temperature for phase transition. At this temperature, the deformed shape-memory alloy returns to its original memorized flat shape, as shown in Fig. 2c (Supporting Information Movie S1 shows the movement of the shape-memory alloy attached to the solar cell).

This dependence of the temperature on the AOI provides an important characteristic for solar tracking. When the perpendicular direction is poorly illuminated and the AOI is low, the shape-memory alloy starts to flatten, and this effect spreads to the alloy attached to adjacent cells, so that a larger part of the solar-cell array becomes aligned perpendicular to the sunlight. The rate of the change in temperature is also important, to respond to the constant movement of the sun and the requirement for continuous shape transformation. As shown in Fig. 2d, the time taken from near-room-temperature to As for the alloy is less than one minute, and this decreases to less than 30 s when the AOI is less than 20°. Considering the speed of the sun’s movement, this rate of heating is sufficient for solar tracking. In addition, cooling to the original temperature under zero illumination takes less than 20 s, so the shape-recovered part of the shape-memory alloy does not hinder the shape transformation of the solar cells overall. The temperature changes under stepwise changes in the AOI are shown in Fig. 2e; they confirm the rate of the change in temperature, with a rapid drop in temperature when the AOI increases by 10°.

We verified that the temperature and its rate of change are sufficient by unfolding tessellated solar cell arrays linked with shape-memory-alloy components under solar simulators, as shown in Fig. 2f (Supporting Information Movie S2 and S3). Solar-cell arrays constructed of both rectangular and triangular units flattened within 1 min under illumination. This confirmed that the heat provided an energy source that was sufficient to actuate the solar cells without an external mechanical input.

**Figure 3:**
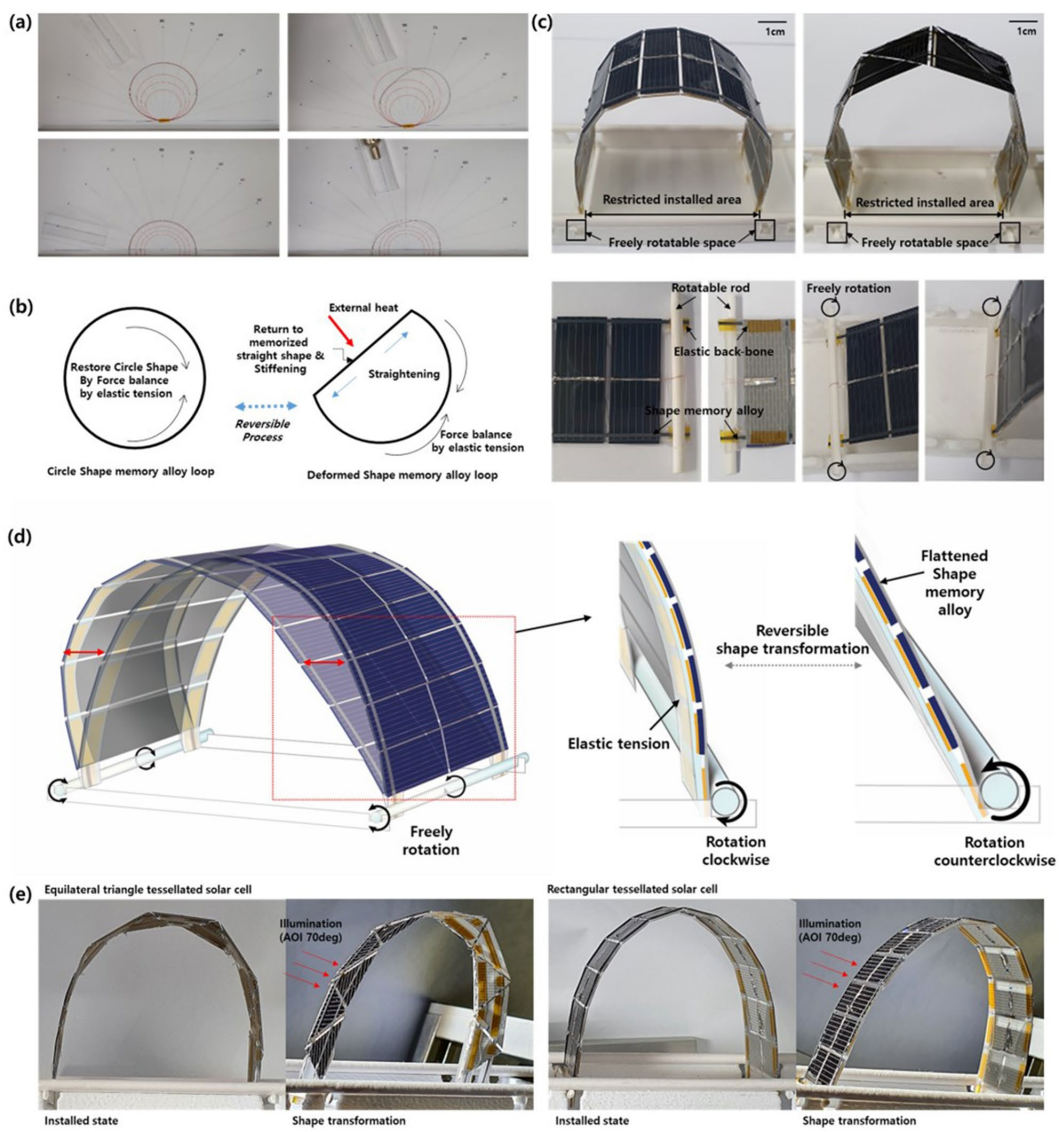
(**a**) Photographs of the wire-type shape-memory-alloy’s shape transformation in response to an external heat source according to the direction of heating, and (**b**) schematic illustration of the transformation mechanism using elastic tension and straightening of the memorized shape of a fixed loop and arch shape. (**c**) Photograph of an installation of rectangular (top left) and right-angled (top right) 2D tessellated solar cells in a restricted area; the front and back sides of the edge part are fixed by a rod (bottom right) to retain the array within a restricted area with free movement (bottom left). (**d**) Schematic illustration of shape transformation within a restricted installation area by free rotation allowing flattening of the surface of the module and recovery to the arch shape by the elastic backbone. (**e**) Photographs of the shape transformation of equilateral-triangle and rectangular tessellated solar cells at an AOI of 70°.

Even though shape-memory-alloy components can actuate a solar-cell array for shape transformation under illumination, this process is generally irreversible. Solar tracking requires that the array returns to its original shape when not illuminated. There are a number of methods for inducing reversible actuation of NiTi-based shape-memory alloys, such as counter loads. In this study, we utilized the shape-memory-alloy loop concept, which has been used in shape-memory alloy motors to create reversible actuation^21–23^. When a shape-memory alloy with a memorized straight-line geometry is formed into a loop, circular geometry is maintained due to the balance of elastic tension. When part of the loop is heated by an external source to above the phase transition temperature, the heated part becomes straight and the stiffness or elastic modulus increases, inducing distortion of the circle. The heated part will then be aligned perpendicular to the heating source, as shown in Fig. 3a,b. When the external heat source is removed, the straightened part undergoes phase recovery to the soft martensite phase, and the loop returns to its original circular geometry to balance the line tension. The same process can be observed in arch-shaped or semicircular geometry when the end points are fixed but are free to rotate.

In this study, we investigated an arch-shaped automated shape-transformable tessellated solar-cell module, as shown in Fig. 3c. To maintain the arch shape without illumination, a soft elastic backbone such as silicone rubber or copper mesh was placed beneath the tessellated solar-cell units and the shape-memory-alloy components were attached to the surface of the solar cells, as shown on the left side of the bottom photograph in Fig. 3c. Within the arch shape, each end of the solar-cell array was designed to rotate freely but to be restricted in lateral or vertical displacement to remain within the installation area, as shown on the right side of the bottom photograph in Fig. 3c,d. In a restricted installation area, when part of the array is heated by incident sunlight and the heat is transferred to the shape-memory-alloy components. The surface temperature of shape-memory-alloy increased with the transferred heat, and it changes to a flattened memorized shape due to the phase transition. According to changed shape-memory-alloy, the freely rotation part rotates counterclockwise to create the shape-changed arch to flattened and with that flattened force, opposite side of arch relatively more curved in installed area. When the illumination is removed, the original arch shape is recovered by the elasticity of the backbone through free rotation counterclockwise. Using such free rotation in a restricted area, it is possible to transform the array into a range of shapes. Based on the arch shape and the range of tessellation units available, a wide range of extended designs can be created, for example 3D dome shapes. However, in this study, simple 2D arch shapes were investigated to verify their solar-tracking performance.

The shape changes in the arch-shaped solar-cell array at an AOI of 70° under 1 sun are shown in Fig. 3e (a photograph of the transformation of the right-angled-triangle tessellated solar cell array is shown in Figure S1, and videos of the transformation are provided in the Supporting Information as Videos S4, S5 and S6). The shape of the tessellated solar-cell array varies such that a greater part of the arch is aligned perpendicular to the direction of the incident light, forming a larger receiving area. In addition, under illumination from a perpendicular direction (AOI 0 degree), the upper part of the arch becomes flattened and the arch decreases in height to receive vertical illumination over a larger area, as shown in Figure S2. The unit shapes used in the shape-transformable tessellated solar-cell arrays have only a limited effect on the solar-tracking shape transformation overall.

**Figure 4:**
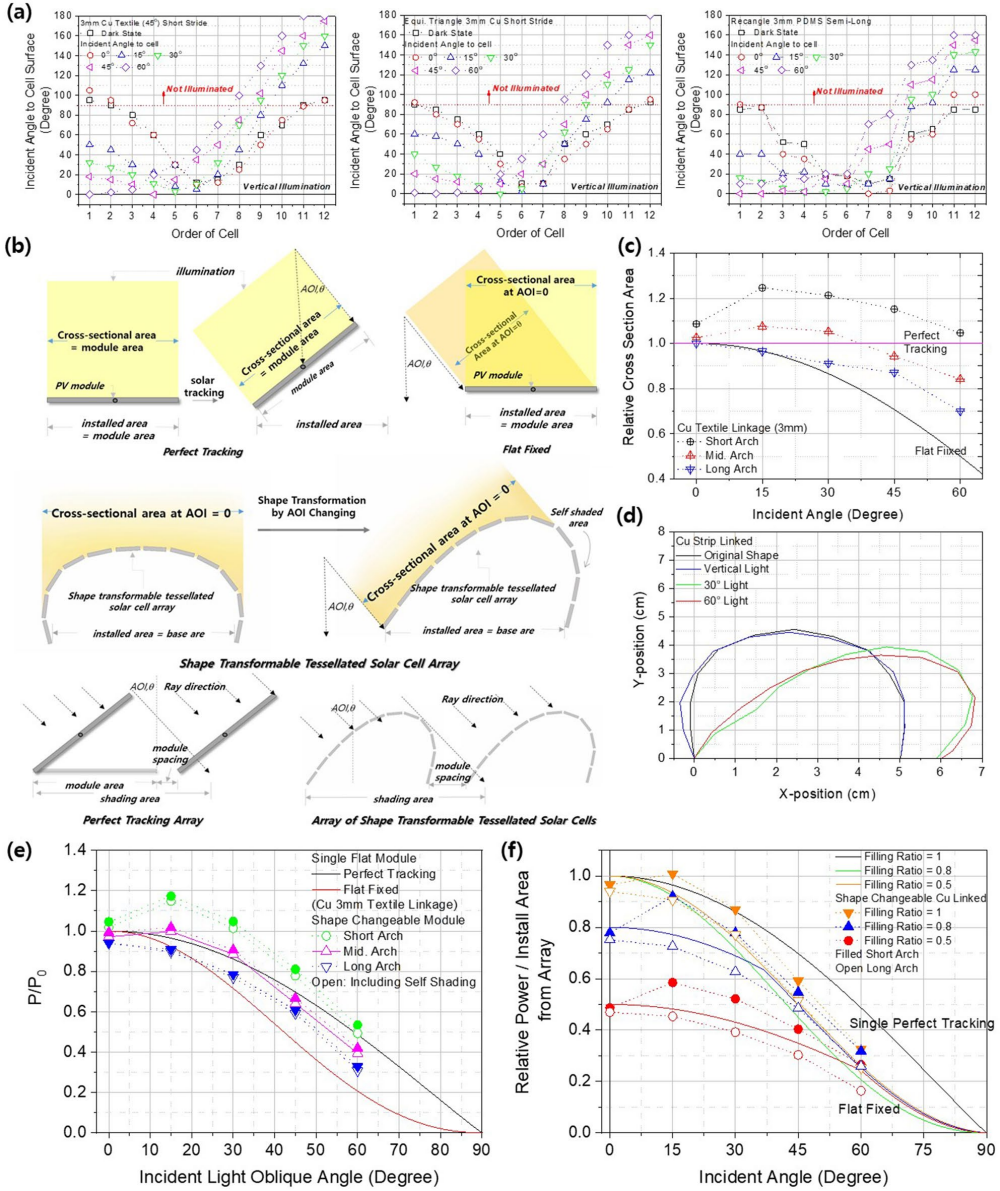
(**a**) Solar-tracking performance of a 2D tessellated solar-cell array according to the AOI and type of backbone. Illustration (**b**) and simulated values (**c**) comparing cross-sectional areas of a fixed flat perfect-tracking system and transformation of the arch-shaped tessellated solar cell array depending on AOI. (**d**) Actual arch geometry simulation results according to the AOI. (**e**) Simulation power-production results comparing a perfect tracking system and the transforming 2D arch-shaped tessellated solar cell array according to the AOI and (**f**) the interval between modules.

The solar-tracking performance of the tessellated shape-transformable solar-cell arrays was analyzed quantitatively by measuring the AOI of each tessellation unit in the array using a solar simulator, as shown in Fig. 4a. For vertically incident light, the cells located in the middle of the array rotated slightly towards the incident direction, and with a further increase in the AOI, at least half of the cells had an AOI of less than 30° relative to the light source. One feature of interest is that cells on the side of the tessellated array opposite to the incoming light have an AOI of greater than 90°, which means that they are directed towards the ground, i.e., they are self-shadowing. They can therefore receive light reflected from the ground or surroundings, similar to bifacial photovoltaic modules. A tessellated array constructed of equilateral triangle units exhibited similar behavior to that of an array constructed of rectangular units. However, an array constructed of right-angled triangles exhibited a characteristic feature in that panels moved in pairs relative to the incident light, so that two panels simultaneously had similar AOIs.

Solar-tracking performance can be estimated quantitatively from the geometry of the arch-shaped tessellated array and the AOI of illumination by calculating the cross-sectional area relative to the incident sunlight, as illustrated in Figureb, and comparing this to the case of perfect tracking of a flat, fixed two-dimensional cell. For perfect tracking, with a given photovoltaic area, the area cross-sectional to the incident beam is identical to the original area, and the ratio between these areas is 1 for all AOI values. For flat modules fixed to the ground, the cross-sectional area decreases with increasing AOI, such that the ratio of the cross-sectional area to the installed area follows the cosine of the AOI. In Fig. 4c, these cases are compared to the calculated cross-sectional area of the shape-transformable tessellated solar-cell array based on the measured AOI of each cell unit. When the arch geometry is high and short, the relative cross-sectional area of the incident beam to the installed area is larger than that of the perfect tracking case until the AOI reaches 60°. With increasing arch width and decreasing height, the relative cross-sectional area decreases and approaches that of the flat fixed case, as expected from the geometric changes. This result shows that, even for a simple arch shape, a shape-transformable tessellated solar-cell array can exhibit more effective solar-tracking performance than perfectly tracking solar cells. This is mainly due to the larger surface area compared to the installed area, as shown in Fig. 4d, which shows the actual arch geometry obtained from the measured AOI of each constituent cell. For the incident beam, as designed and expected, the illuminated part of the array flattened through flattened actuator, pushing the opposite side to create more curved shape (Figure S1) and inducing a larger light-receiving area. Even under vertical illumination, the middle part of the array flattened and the total shape widened like a pot as shown in Figure S2, exceeding the installed area by depressing the arch to receive more light.

Based on the relative cross-sectional areas of the incident light, we estimated the power production of shape-transformable tessellated solar-cell arrays, a perfect tracking module and a flat fixed module, considering the effects of light intensity and AOI on the power production of the crystalline Si solar cells (detailed calculation procedures are described in the Supporting Information). To calculate the effect of light intensity, a single diode model was used, based on parameters obtained from fitting the voltage-current density curves measured for the PERC cells used in this study. The effect of the AOI on PERC-type solar cells was obtained from our previous work^16,18^. Combining the effects of the cross-sectional area, the AOI and the light intensity, Fig. 4e shows the expected power relative to that from vertical 1-sun illumination over the installed area for a single module. The expected power output of a shape-transformable tessellated solar-cell array with a short and high arch structure exceeded those of the perfect-tracking and fixed-flat cases.

Shape-transformable solar-cell arrays do not require any additional energy or machinery, and can be installed in restricted spaces in urban environments. When scattered light incident on self-shaded areas is considered, the power enhancement increases further. Hence, shape-transformable tessellated solar-cell arrays can exhibit better performance than perfect tracking systems, as shown in Fig. 4f. When there are large numbers of installed modules, mutual shading of and by modules can limit performance during tracking, as shown in Fig. 4b. Therefore, if the spacing between modules is insufficient, shaded modules cannot operate at full capacity. This presents a significant limitation to utilization of tracking systems at the plant level. However, shape-transformable tessellated solar-cell arrays demonstrate improved performance in this regard by reducing the shadowed area. Based on the calculations, it was therefore anticipated that shape-transformable tessellated solar-cell arrays would provide performance superior to that of perfect-tracking solar modules in both urban and plant applications.

**Figure 5:**
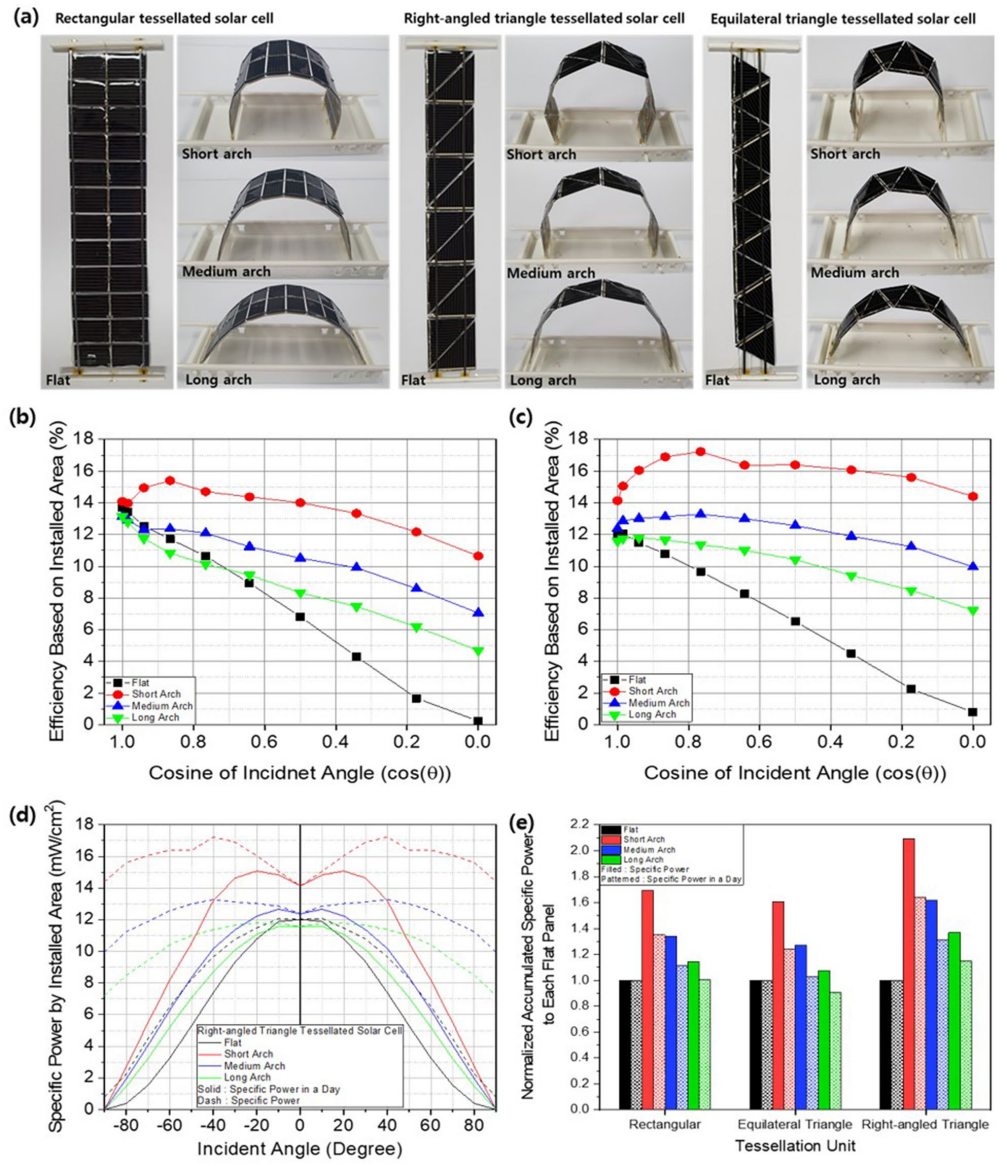
(**a**) Photographs of rectangular, right-angled and equilateral-triangle-shaped tessellated solar cells in flat and various 2D arch states in a restricted installation area. Efficiency based on installed area of arches comprised of (**b**) rectangular and (**c**) right-angled-triangle 2D tessellated solar cells. (**d**) Rectangular 2D tessellated solar cell’s accumulated power and power over a day and (**e**) relative accumulated power and power over a day compared according to a restricted installation area for flat and various 2D arch shapes.

These predictions were verified by measuring the power output of shape-transformable 2D tessellated solar-cell arrays consisting of 12 rectangular, right-angled-triangle and equilateral-triangle units, as shown in Fig. 5a. Efficiency was calculated based on the maximum power output from the array per unit installed area, which was defined as the base area of the arch shape (definition of based or installed area is shown in Figure S3). Each cell in the array was electrically interconnected in a mixed parallel and series configuration. The measured output power of the tessellated solar-cell arrays (shown in Supporting Information Figure S4) decreased with increasing AOI or decreasing cosine of the AOI in all forms of flat fixed tessellated solar-cell arrays. However, the superior solar-tracking performance of the shape-transformable 3D tessellated solar-cell arrays was little affected by the AOI (Fig. 4e). In the case of assessing only power output, the installed area was not considered. Considering the installed area, shown in Fig. 5a, the efficiency of shape-transformable tessellated solar-cell arrays with rectangular units is shown in Fig. 5b and that of right-angled triangular units is shown in Fig. 5c. (The performance of arrays using equilateral-triangle tessellated solar-cell arrays is shown in Supporting Information Figure S5). The installed-area-based efficiency was enhanced by shape-transformable solar tracking in all cases. A tall and narrow arch demonstrated superior solar-tracking performance, resulting in greater efficiency for a given installed area with little effect of AOI on electricity production. Both shapes of triangular units exhibited better performance than that of the rectangular unit. This implies that the shape of the integrated units affects performance through shape transformation efficiency, and that additional experiments with other shapes may provide further performance enhancements.

The efficiency of shape-transformable tessellated solar-cell arrays with respect to the installation area can provide superior omnidirectional performance compared to flat fixed solar panels. To consider actual electricity production per day, the cosine law of the AOI relative to solar illumination was applied to the power production according to the AOI as shown in Fig. 5d (see Supporting Information Figure S6 for solar-cell arrays comprised of units of other shapes). The power relative to the installed area decreased with increasing AOI due to the decreased light intensity at increased AOIs. However, the power produced by the shape-transformable 3D tessellated solar-cell arrays was greater than that of flat fixed solar cells at all AOIs. This relationship was clear when comparing the accumulated power per installed area, as shown in Fig. 5e. Among the shapes of tessellated solar cell, right-angled triangle shape showed best performance in short arch. In our opinion, two right-angled triangles form a square as a pair and this one pair receives almost the same amount of light. So it is considered that the loss is minimum from very slight difference in incident light intensity in right-angled triangle tessellated solar cell. Without considering changes in light intensity due to the AOI, the shape-transformable tessellated solar-cell arrays exhibited a twofold increase in electricity production for the same installed area. Including the effects of changes in light intensity arising from changes in the AOI, which more closely represents actual solar illumination conditions, the electricity production of the shape-transformable solar-cell array constructed of right-angled triangular units was 60% higher than that of a flat fixed solar-cell array.

Shape-transformable tessellated solar-cell arrays can enhance electricity production beyond what is possible even with perfectly tracked flat solar panels through superior performance under omnidirectional incident light. In particular, this system overcomes the limitations of conventional tracking systems in urban environments, such as limited installation areas and the need for complex machinery for actuation. In addition, the part of the array that is self-shaded during shape transformation acts as the reverse side of a bifacial photovoltaic module, providing shape-transformable tessellated solar-cell arrays with the advantages of both a solar-tracking system and a bifacial PV module. This study introduces the shape-transformable photovoltaic module concept; many more areas of research remain, including efficient power management of each cell and 3D designs suitable for specific applications.

In particular, a range of possible tessellation unit geometries will be explored, offering the possibility of 3D structures with solar-tracking shape transformation alongside other functionalities or aesthetic characteristics suitable for specific applications.

## Discussion

In this study, a new concept of automated solar tracking using shape-transformable 3D tessellated solar-cell arrays was introduced, based on tessellated solar-cell units and the use of shape-memory alloys to actuate movement of the units in response to the heat of sunlight on the surface of the array. The array changes its shape to present a larger cross-sectional area to incident sunlight by using shape-memory-alloy actuation in conjunction with an arch-shaped array with fixed displacement and freely rotating ends. The arch-structured transformable tessellated solar-cell arrays demonstrated solar-tracking performance superior to that of perfect-tracking photovoltaic modules and had the additional advantage of being bifacial solar modules in self-shaded locations. Their superior solar-tracking performance resulted in enhanced power production compared to conventional solar-tracking systems or fixed solar cells. Considering the movement of the sun, over a day, this system provides 60% more electricity. In addition, using various designs of tessellation units, dome-shaped or other 3D structures could be designed that are suitable for specific applications or to meet aesthetic requirements.

## Methods

### Fabrication shape transformation 3D tessellated solar cell

For the tessellated solar-cell units, passivated emitter rear cells (PERCs; LWM5BB-210, Lightway) were used after cutting them into right-angled triangle, equilateral-triangle and rectangular shapes. Each solar-cell array was constructed of 12 units arranged with uniform spacing and fixed on a backbone strip using 135-μm-thick insulating tape and silicone pressure-sensitive adhesive (Youngwoo). The elastic backbone was made of 1-mm-thick silicone rubber and polydimethylsiloxane (PDMS; Sylgard 184 A/B, Heesung STS) or copper (Cu) mesh (120 Mesh, 0.131 opening size, Hyunjin Mesh Filter). The solar cells were interconnected with commercial Cu wire (100 μm) or Cu mesh with soldering. Pb-free wire (HSE-02-SR34, Heesung Material LTD.) and a soldering iron (FX-951, Hakko) were used for soldering. After electrical connection, the shape-memory-alloy components (Nitinol Flat Wires, 45 °C, Kellogg’s Research Labs) were attached to the surface of the solar cells using insulating tape.

After assembling the tessellated solar cells and the shape-memory-alloy components, the modules were encapsulated with a PDMS silicone material (Sylgard 184 A/B, heesungSTS) using a casing method. A 1:1 (A:B) ratio mixture was poured onto the surface of the tessellated cells then cured at 70 °C in a convection oven.

### Characterization

Differential scanning calorimetry (DSC; Q20, TA Instruments) was conducted from 20 °C to 100 °C at a heating/cooling rate 10 °C/min to establish the thermal properties of the shape-memory alloy. To measure the temperatures of the surface of the solar cell and of the shape-memory alloy at a distance of 3 mm from the surface of the array, a thermometer (mini Logger GL840, Graphtec) was used. To measure the photovoltaic performance of the self-solar-tracking tessellated solar cells and characterize the shape transformation, we first calibrated a solar simulator (Sun 2000, 1000 W Xenon source; Abet Technologies; 2400 Keithley source meter) using a KG-3 filter and an NREL-certified reference cell, and then set the simulator to 1 sun, 1.5 AM conditions. For the estimation of photovoltaic performance, we have measured specific power based on installed area and to consider the performance a day, we estimated it according to AOI effect. The sample stage was rotated at intervals of 10° from 0° to 90° and we used the fabricated stage that can rotate at the exact angle through the motor and sensor.

## Supplementary Information

Supplementary Information 1.

Supplementary Video 1.

Supplementary Video 2.

Supplementary Video 3.

Supplementary Video 4.

Supplementary Video 5.

Supplementary Video 6.

Supplementary Information 2.
