# Supplementary material for: Automated shape-transformable self-solar-tracking tessellated crystalline Si solar cells using in-situ shape-memory-alloy actuation
Source: Sci Rep. 2022 Jan 31;12:1597. doi: 10.1038/s41598-022-05466-7 (PMC8803980; doi:10.1038/s41598-022-05466-7)
Supplement: Supplementary file 1 — Supplementary Information 1. [file 41598_2022_5466_MOESM1_ESM.docx]

**Legends for video files (Supporting Information)**

Movie S1. Shape transformation of SMA integrated with 2-solar cell.

Movie S2. Shape transformation of folded 5 equilateral triangle shaped solar cells with SMA.

Movie S3. Shape transformation of rolled 6 rectangular shaped solar cells with SMA.

Movie S4. Automated shape transformation of small arch structured with rectangular shaped solar cell array under AOI 70 degrees.

Movie S5. Automated shape transformation of small arch structured with right-angled triangle shaped solar cell array under AOI 70 degrees.

Movie S6. Automated shape transformation of small arch structured with equilateral triangle shaped solar cell array under AOI 70 degrees.
