# Supplementary material for: Automated shape-transformable self-solar-tracking tessellated crystalline Si solar cells using in-situ shape-memory-alloy actuation
Source: Sci Rep. 2022 Jan 31;12:1597. doi: 10.1038/s41598-022-05466-7 (PMC8803980; doi:10.1038/s41598-022-05466-7)
Supplement: Supplementary file 8 — Supplementary Information 2. [file 41598_2022_5466_MOESM8_ESM.pdf]

Automated Shape-Transformable Self-Solar-Tracking Tessellated Crystalline Si Solar Cells using In-Situ Shape-Memory-Alloy Actuation

Min Ju Yun[1], Yeon Hyang Sim[1,2], Dong Yoon Lee[1] and Seung I. Cha*[1,2]

1. Energy Conversion Research Center, Electrical Materials Research Division, Korea Electrotechnology Research Institute

2. Department of Electro-functionality Materials Engineering, University of Science and Technology

Supporting Information

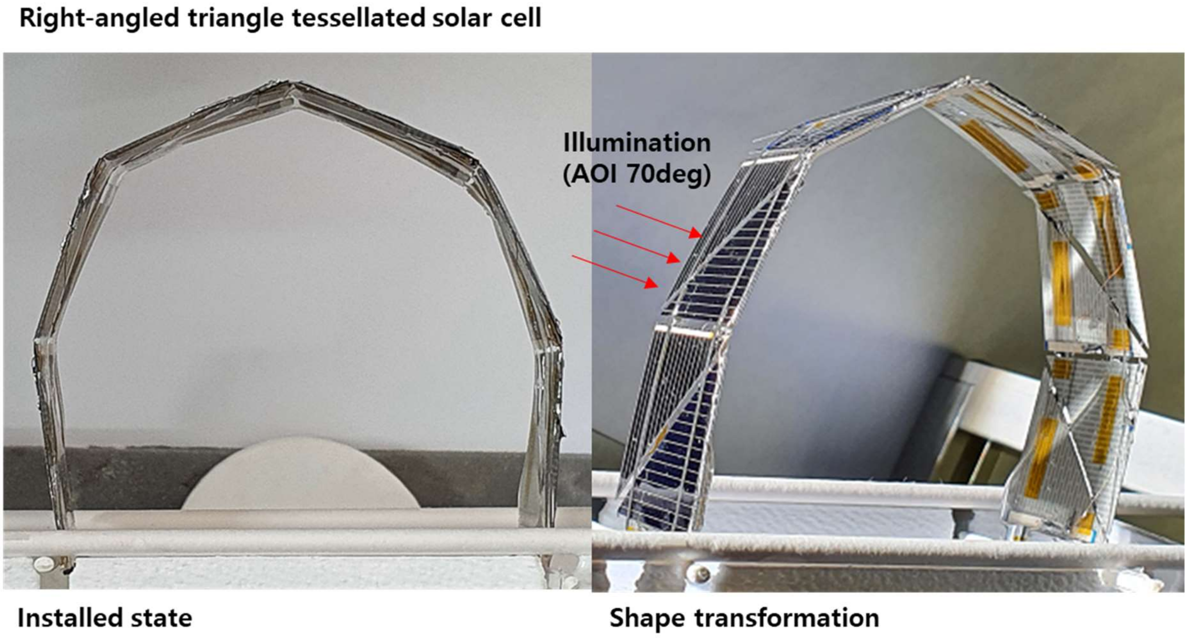

Figure S1. Photographs of right-angled triangle tessellated solar cell module's initial state and shape transformation at AOI 70 degree.

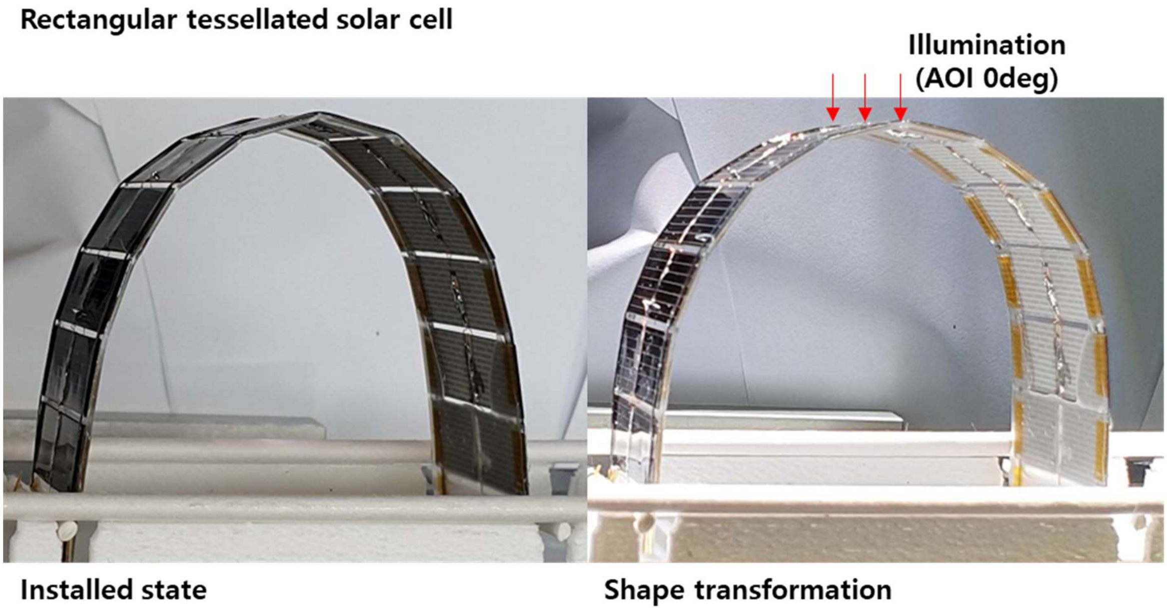

Figure S2. Photographs of rectangular tessellated solar cell module's initial state and shape transformation at vertical illumination (AOI 0 degree).

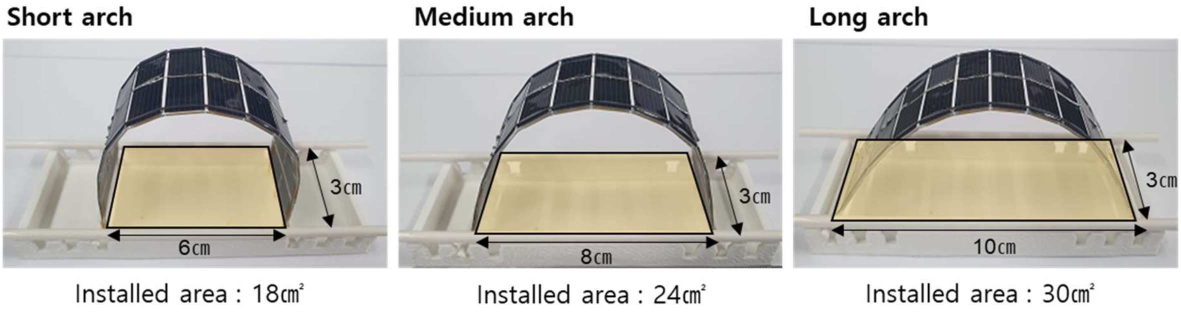

Figure S3. Photographs of definition of based or installed area depending on arch length.

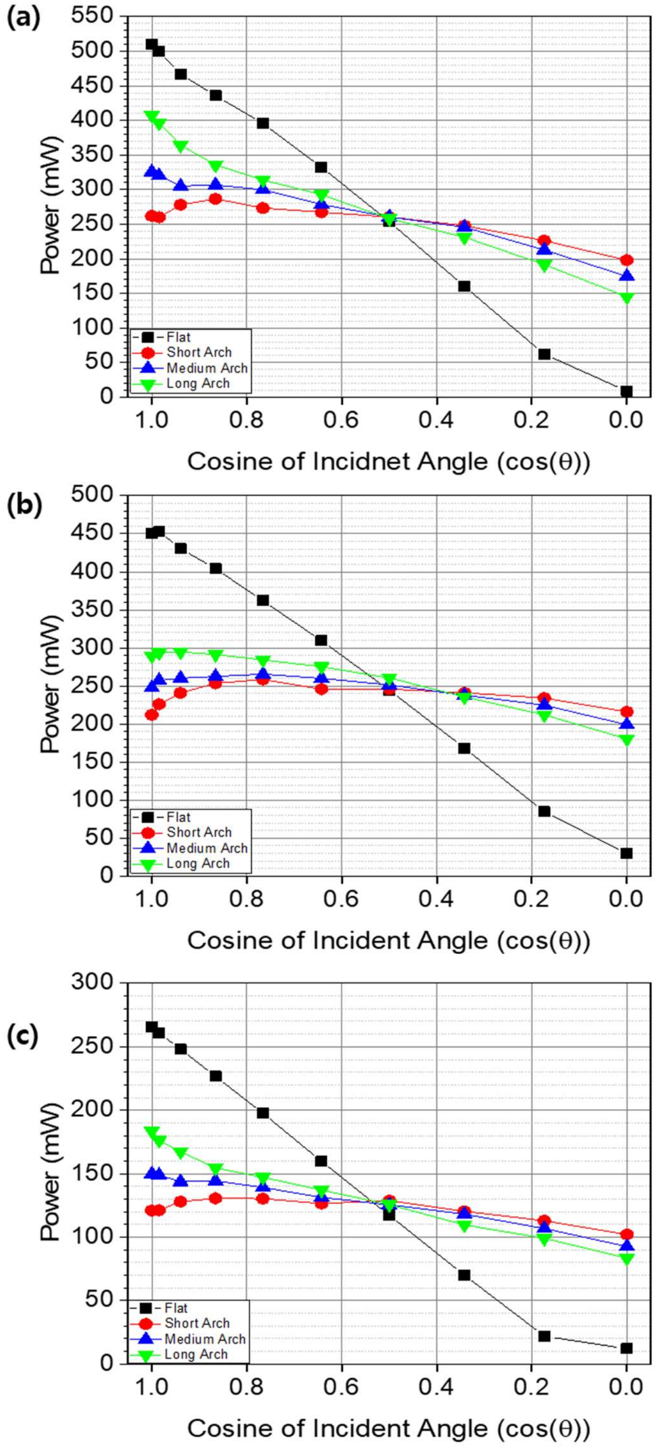

Figure S4. Power output of (a) rectangular, (b) right-angled and (c) equilateral triangle shape tessellated solar cell module according to AOI.

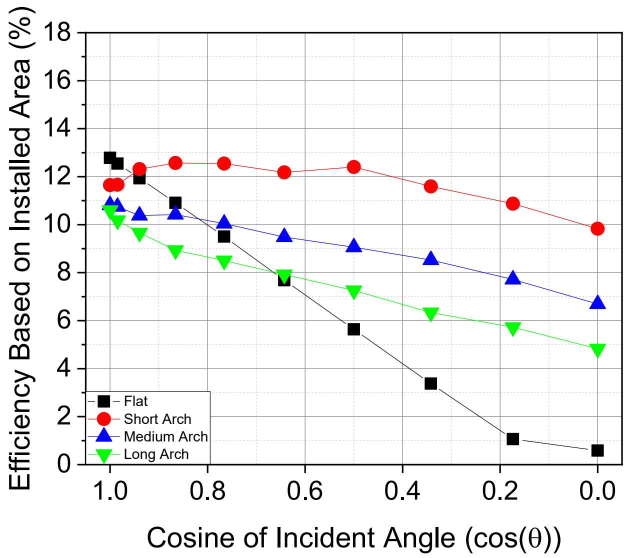

Figure S5. Maximum power output considering installed area forming arch geometry of equilateral triangle tessellated solar cell.

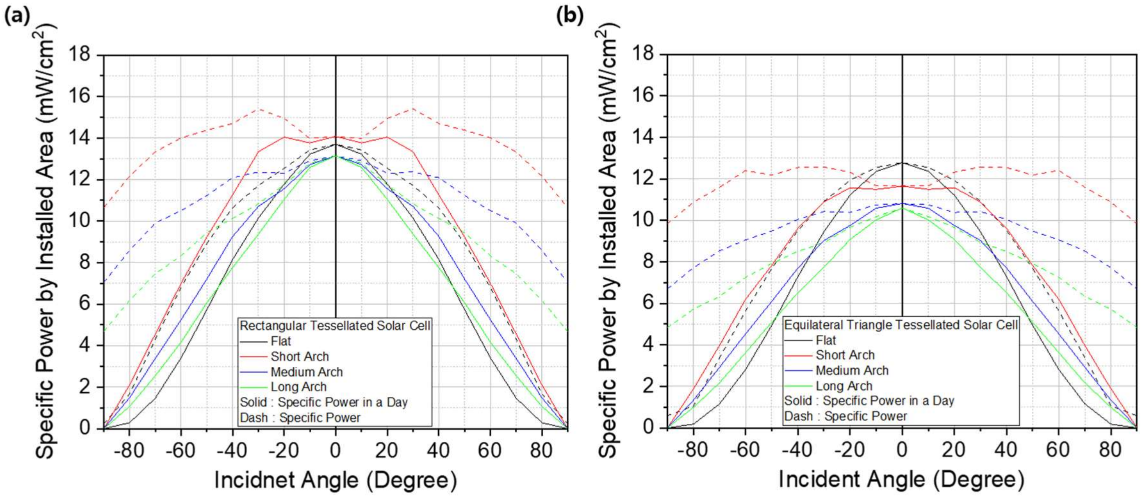

Figure S6. Accumulated power and power in a day of (a) right-angled and (b) equilateral triangle tessellated solar cell.

Calculation Details for Figure 4.

Terminology and Basic Parameters

- Incident light intensity: I
- 2 dimensional module area: l
- Incident light intensity according to sun altitude (θ): $I = I_0 \cos\theta$
- Relative light intensity in shade: I_{sh}
- (I_0 = light intensity of normal incident of 1sun, 1.5 A.M. condition)
- Cross sectional area: A_c
- Shaded area on module: $A_l = l \ \frac{X_s}{X_t}$
- Actual light intensity (when partially shaded): $\mathrm{I} = I_0 \cos\theta \frac{A_c}{l} + I_0 I_{sh} \frac{A_l}{l} = \frac{I_0}{l}(A_c \cos\theta + A_l I_{sh})$
- Power production from module = $P = I \ \ l \ \ \eta$ where η=power conversion efficiency
- Power production from reference condition = $P_0 = I_0 l \ \ \eta_0$ where η_0 is power conversion efficiency under vertical illumination at I_0.
- Effect of light intensity and angle of incident (AOI) on power production = $P = I \ \ l \ \ \eta = I \ \ l \ \ \eta_0\eta_\theta\eta_I$ where η_θ is effect of AOI on efficiency and η_I is effect of relative light intensity to I_0. (assumption)
- Shading area = $S_A = l \ \ \cos\alpha + h \ \ \tan\theta = l \ \ \cos\alpha + l \ \ \sin\alpha \tan\theta \ +$. If perfect tracking, α = θ and $S_A = \frac{l}{\cos\theta}$.

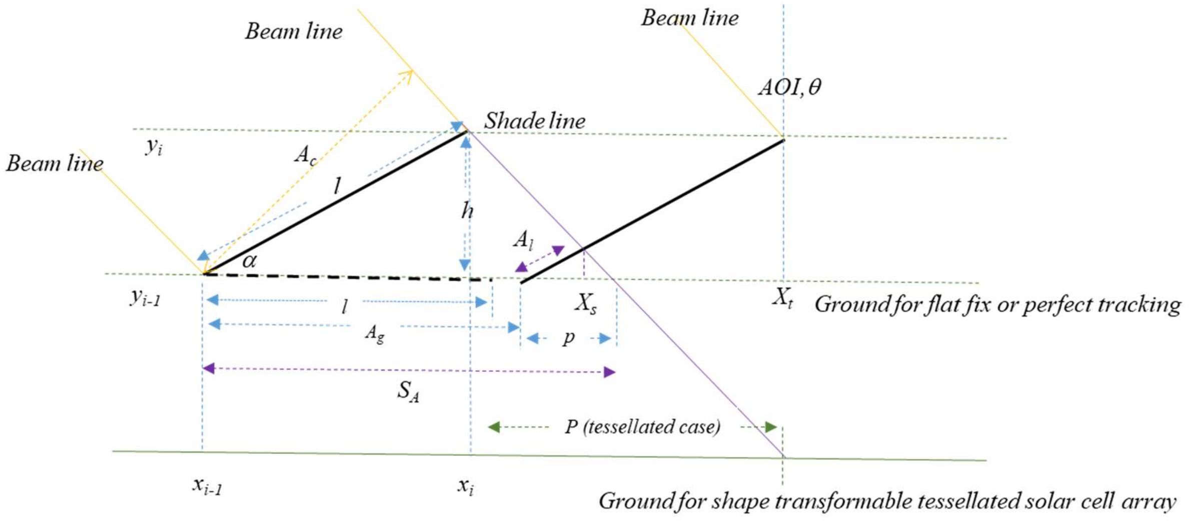

Effect of Relative Light Intensity and AOI on Power Conversion Efficiency

- In crystalline Si solar cells, open circuit voltage changes with current density, i.e. incident light intensity. Therefore, the power output changed additionally to the linear relationship with light intensity. If light intensity of incident light is βI_0 where β is ratio between actual light intensity and reference intensity, the power output = $\beta J_0 l \ \ V(J)$ where j_0 is current density at reference light intensity.
- By single diode model, $V(J) = \frac{nkT}{q}\ln\left(\frac{J_0}{a_0}\beta + 1\right) = \mu\ln(\gamma\beta + 1)$ where a_0 is dark current and γ, μ are constant. To obtain these constant values, we have measured the voltage at 1 sun condition and fitting the curves and find the value of μ=0.0258519 and γ=1.48× 10^{11} at 300K temperature.
- Considering relative power, $\frac{P}{P_0} = \frac{\beta J_0 l \eta_0 \eta_I}{J_0 l \eta_0} = \frac{\beta J_0 l \mu \ln(\gamma\beta+1)}{J_0 l V_0}$, the efficiency changes by light intensity obtained as $\eta_I = \frac{\mu l \ \ (\gamma\beta+1)}{V_0} = \mu' \ln(\gamma\beta + 1)$ where μ'=0.03887504 from measured data.
- The effect of power output by AOI changes were measured. It is forms of linear relationship to cosine of incident angle as $\eta_\theta = (1 - B) + B\cos\theta$, where B is obtained as 0.3 for PERC cell in fitting with measured data.

Flat Fixed Solar Modules

- For flat fixed solar module, the $\alpha = 0$ in Figure S1. Therefore, the power output can be obtained as $P = IA_c\eta = I_0\cos\theta\, l\cos\theta\, \eta_0\eta_I\eta_\theta = P_0 cos^2\theta\mu\ln(\gamma\cos\theta + 1)\,(1 - B + B\cos\theta)$.
- Therefore, the power output ratio to reference state, generated by AOI, is give as $\frac{P}{P_0} = cos^2\theta\mu\ln(\gamma\cos\theta + 1)\,(1 - B + B\cos\theta)$.
- For the array of fixed flat modules, there are no need for considering shadows. Therefore, just considering the filling ratio of flat fixed solar modules, the power output for array can be obtained.

Perfect Tacking Solar Modules

- For perfect tracking solar module, the $\alpha = \theta$ in Figure S1 and AOI to incident angle is always 0. Therefore, the power output can be obtained as $P = IA_c\eta = I_0\cos\theta\, l\eta_0\eta_I = P_0 cos\theta\mu\ln(\gamma\cos\theta + 1)$.
- Therefore, the power output ratio to reference state, generated by AOI, is give as $\frac{P}{P_0} = cos\theta\mu\ln(\gamma\cos\theta + 1)$.
- For the array of perfect tracking, the shadows affect the power output should be considered. If perfect tracking modules installed with spacing between them to avoid shadowing, the filling ratio can be defined as $R = \frac{l}{A_g}$ in Figure S1. Then the shading area in ground can be obtained as $p = S_A - A_g = \frac{l}{\cos\theta} - \frac{l}{R}$. Therefore the shading area can be calculated as $A_l = p\cos\theta = l\left(1 - \frac{\cos\theta}{R}\right)$.
- In this case the light intensity changed by shading as $I_s = I(l - A_l) + I_0 I_{sh} A_l = I_0 l\left(\frac{cos^2\theta}{R} + I_{sh}\frac{R-\cos\theta}{R}\right) = I_A I_0 l$, where $I_A = \left(\frac{cos^2\theta}{R} + I_{sh}\frac{R-\cos}{R}\right)$. Therefore, the shaded modules produce the power as $P_s = P_0 I_A \mu\ln(\gamma I_A + 1)$.

- For array of N modules, first module is not affected by shadows. Therefore, total power can be obtained as $P_{array} = P_0(cos\theta\mu \ln(\gamma \cos\theta + 1) + (N-1)I_A\mu \ln(\gamma I_A + 1))$. Therefore installed area efficiency can be calculated as $\frac{P_0(cos\theta\mu \ln(\gamma \cos\theta+1)+(N-1)I_A\mu \ln(\gamma I_A+1))}{NA_g}$.

Shape Transformable Tessellated Solar Cell Arrays

- Tessellated solar cell array consists of several cells with different α and we should consider x and y coordination to obtain cross-sectional area, shading length, power generated by arrays. In addition, without measuring α of each unit, other properties could not be obtained.
- For each unit, cross sectional area ($A_{c,i}$), power output (P_i) , x of end point (x_i) and y of end point (y_i) should be calculated and summation for all units to obtain total value of array. But, when the AOI exceeds 90 degrees, i.e. self-shading, cross sectional area and power output must be considered separately. When power from shadows is not considered, the power output from units with AOI over 90 degrees is 0. However, shadow has certain amount of light scattered from surrounding, it produces some amount of power which is main source for bifacial-like effect.
- For i[th] unit in array, the cross-sectional area from Figure S1 can be calculated as $A_c^i = l\cos(\theta - \alpha)$ and total cross-sectional area can be obtained as $A_c = \sum_i^n A_c^i$ for $A_c^i > 0$.
- The power output for each unit can be obtained as $P^i = I_0 \; l\cos(\theta-\alpha)\,\eta_0\mu\ln(\gamma\cos(\theta-\alpha)+1)(1-B+B\cos(\theta-\alpha) = IP_0 \; \cos(\theta-\alpha)\,\mu\ln(\gamma\cos(\theta-\alpha)+1)(1-B+B\cos(\theta-\alpha)$ if AOI for each unit $(\theta-\alpha)$ is less than 90 degrees. If considered self-shading power $P^i = P_s$ for self-shaded unit. Therefore, total power can be obtained by summation of P^i.
- For location of each unit can be calculated as $x_i = x_{i-1} + l\sin\alpha$ and $y_i = y_{i-1} + l\cos\alpha$ where $x_0, y_0 = 0$. The installed area of array can be obtained by $A = \sum_1^n x_i$.
- The shadow area by each unit can be obtained by $S_a^i = x_i + y_i\tan\theta$ in geometric consideration. Total shading area from the shape transformable tessellated solar cell array is given by the maximum value from the calculated S_a^i from each unit, i.e. $\max(S_a^i)$. The shadow edge is calculated when S_a^i becomes maximum from calculated S_a^i of each unit. The location of shadow edge on next module in arrays can be obtained by following algorithm.

  ① Obtain shadow edge line equation as $y = -\frac{x}{\tan\theta} + \frac{p(tessellated)}{\tan\theta}$.

  ② Obtain line equation of each unit as $y = x\tan\theta + y_i - x_i\tan\theta$

  ③ Obtain cross point between above two lines as $(x_{s,i}, y_{s,i})$.

  ④ If $(x_{s,i}, y_{s,i})$ is between (x_{i-1}, y_{i-1}) and (x_i, y_i) then, the shadow edge is in i[th] unit and j[th] unit where j<i, is fully shadowed and i[th] cell has light intensity of $I = I_0\cos\theta\frac{x_i - x_{s,i}}{x_i - x_{i-1}} + I_0 I_{sh}\frac{x_{s,i} - x_s}{x_i - x_{i-1}}$ and utilizing A_c, η_i for this value and η_θ.

  ⑤ Total power can be obtained by summation of each P_i.

  ⑥ For several arrays, first array has no shading effect and other arrays have shadowing effect.

Power from fully shadowed cells

- If shadowed cell or unit has received light by scattering and reflection, this light incident with isotropic AOI. Therefore, if shadowed area has relative light intensity of $I = I_0 I_{sh}$, power can be obtained by $P_{sh} = P_0 \eta_i (I_0 I_{sh}) \eta_\theta$.
- In order to obtain η_θ, isotropic AOI should be considered as $\eta_\theta = \frac{2\int_0^{\pi/2} \eta_\theta d\theta}{\pi} = \frac{2\int_0^{\pi/2} (1 - B + B\cos\theta)\ d\theta}{\pi} = (1 - B) - 2\frac{B}{\pi}$.
- If $I_{sh} = 0.1, B = 0$, i.e. shadows has 10% light intensity to 1sun direct illumination, $\frac{P_{sh}}{P_0}$ is about 0.0463.
